# Comparison of Unipolar and Bipolar Voltage Mapping for Localization of Left Atrial Arrhythmogenic Substrate in Patients With Atrial Fibrillation

**DOI:** 10.3389/fphys.2020.575846

**Published:** 2020-11-26

**Authors:** Deborah Nairn, Heiko Lehrmann, Björn Müller-Edenborn, Steffen Schuler, Thomas Arentz, Olaf Dössel, Amir Jadidi, Axel Loewe

**Affiliations:** ^1^Institute of Biomedical Engineering, Karlsruhe Institute of Technology (KIT), Karlsruhe, Germany; ^2^Department of Electrophysiology, University-Heart-Center Freiburg-Bad Krozingen, Bad Krozingen, Germany

**Keywords:** atrial fibrillation, bipolar voltage mapping, unipolar voltage mapping, arrhythmogenic substrate, low voltage areas

## Abstract

**Background:** Presence of left atrial low voltage substrate in bipolar voltage mapping is associated with increased arrhythmia recurrences following pulmonary vein isolation for atrial fibrillation (AF). Besides local myocardial fibrosis, bipolar voltage amplitudes may be influenced by inter-electrode spacing and bipole-to-wavefront-angle. It is unclear to what extent these impact low voltage areas (LVA) in the clinical setting. Alternatively, unipolar electrogram voltage is not affected by these factors but requires advanced filtering.

**Objectives:** To assess the relationship between bipolar and unipolar voltage mapping in sinus rhythm (SR) and AF and identify if the electrogram recording mode affects the quantification and localization of LVA.

**Methods:** Patients (*n* = 28, 66±7 years, 46% male, 82% persistent AF, 32% redo-procedures) underwent high-density (>1,200 sites, 20 ± 10 sites/cm^2^, using a 20-pole 2-6-2 mm-spaced Lasso) voltage mapping in SR and AF. Bipolar LVA were defined using four different thresholds described in literature: <0.5 and <1 mV in SR, <0.35 and <0.5 mV in AF. The optimal unipolar voltage threshold resulting in the highest agreement in both unipolar and bipolar mapping modes was determined. The impact of the inter-electrode distance (2 vs. 6 mm) on the correlation was assessed. Regional analysis was performed using an 11-segment left atrial model.

**Results:** Patients had relevant bipolar LVA (23 ± 23 cm^2^ at <0.5 mV in SR and 42 ± 26 cm^2^ at <0.5 mV in AF). 90 ± 5% (in SR) and 85 ± 5% (AF) of mapped sites were concordantly classified as high or low voltage in both mapping modes. Discordant mapping sites located to the border zone of LVA. Bipolar voltage mapping using 2 vs. 6 mm inter-electrode distances increased the portion of matched mapping points by 4%. The unipolar thresholds (y) which resulted in a high spatial concordance can be calculated from the bipolar threshold (x) using following linear equations: *y* = 1.06*x* + 0.26*mV* (*r* = 0.994) for SR and *y* = 1.22*x* + 0.12*mV* (*r* = 0.998) for AF.

**Conclusion:** Bipolar and unipolar voltage maps are highly correlated, in SR and AF. While bipole orientation and inter-electrode spacing are theoretical confounders, their impact is unlikely to be of clinical importance for localization of LVA, when mapping is performed at high density with a 20-polar Lasso catheter.

## 1. Introduction

Atrial fibrillation (AF) is the most common supraventricular cardiac arrhythmia characterized by an irregular heart rhythm and associated with an increased risk of heart failure, stroke, and mortality (Wang et al., 2003; Miyasaka et al., 2005; Go et al., 2014).

Pulmonary veins have been identified as major arrhythmogenic trigger sites for AF. Therefore, their isolation has become a widely used and effective treatment for AF (Haissaguerre et al., 1998). However, additional arrhythmogenic atrial substrate is present in 30–50% of persistent AF patients and may be responsible for the maintenance of the arrhythmia, resulting in increased AF recurrences after pulmonary vein isolation (PVI) in these patients (Verma et al., 2005, 2015). Procedural identification of arrhythmogenic AF sources with rapid, continuous, or repeated rotational activity has revealed their localization within fibrotic regions displaying low bipolar voltage <0.5 mV during AF (Jadidi et al., 2016, 2020; Seitz et al., 2017). Ablation of these atrial AF sources, in addition to PVI, improves the success rate in persistent AF patients from 30 to 50% with PVI only to 70% with additional selective ablation of arrhythmogenic low voltage areas (LVA) (Rolf et al., 2014; Jadidi et al., 2016; Blandino et al., 2017; Seitz et al., 2017).

Atrial arrhythmogenic fibrosis-rich areas are currently identified using imaging or bipolar voltage mapping. However, in addition to the underlying atrial fibrosis that affects the bipolar voltage (peak-to-peak amplitude of the electrogram), the angle of the bipolar recording electrodes (wavefront-to-bipole orientation), the distance between the electrodes and the electrode size may also influence the bipolar electrogram amplitudes (Schuler et al., 2013; Anter et al., 2015; Beheshti et al., 2018; Lin et al., 2018; Gaeta et al., 2019). Therefore, using bipolar electrograms can potentially cause areas of fibrotic and non-fibrotic tissue to be misclassified. On the other hand, unipolar electrogram voltage is unaffected by the catheter orientation and electrode distances. However, the signals are more susceptible to noise and ventricular far-field requiring advanced filtering (Frisch et al., 2020).

We aim to assess the differences in the extent and distribution of atrial LVA when comparing bipolar to unipolar voltage mapping in AF and sinus rhythm (SR). In this work, we evaluate the correlation between the two mapping methods and identify the corresponding unipolar thresholds that yield the highest concordance to the bipolar LVA. Additionally, we examine the impact of (1) the electrode distance, (2) the anatomical region of the left atrium, and (3) the extent of left atrial (LA) low voltage substrate on the correlation between unipolar and bipolar LVA.

## 2. Methods

### 2.1. Study Cohort and Electro-Anatomical Mapping

A total of 28 patients with AF underwent high-density (>1,200 mapped sites per LA and rhythm, mapping density of 17 ± 7 sites per cm^2^ in SR and 22 ± 11 per cm^2^ in AF) voltage mapping using a 20-pole variable (15–20 mm diameter) Lasso-Nav mapping catheter (electrode size: 1 mm; spacing: 2-6-2 mm). The voltage mapping was performed using CARTO-3 (Biosense Webster, Diamond Bar, CA, USA) and carried out in both rhythms SR and AF prior to PVI. Bipolar voltage maps were acquired using all electrode spacings (2 and 6 mm). Patients were mapped first in the rhythm that they presented in and then cardioverted into SR or induced into AF to obtain the second map. 21/28 patients presented with clinical persistent AF, the remaining had SR at presentation. Nine of the 28 (32%) patients underwent a redo AF ablation procedure after a previous PVI procedure. The remaining 19 (68%) patients came for their first AF ablation procedure.

Electrograms recorded >7 mm from the geometry surface were excluded from the analysis to avoid poor contact points. Additionally, points containing only noise or pacing artifacts were removed based on manual assessment. The unipolar signals were processed by the Carto3 software, which uses standard clinical filtering with highpass and lowpass cutoff frequencies at 2 and 240 Hz to remove high and low frequency noise from the acquired EGM. Additionally, a notch filter was applied to clear the noise from the environment power lines. For the unipolar recordings Wilson's Central Terminal (WCT) was used as the reference electrode. Additionally, bandpass filtering was performed at 16–500 Hz for the bipolar signals. A window of interest was defined prior to the QRS complex to identify atrial activity and the voltage provided by CARTO-3 was obtained by taking the peak-to-peak value (local maximum − local minimum) of a single atrial beat in a 2.5 s second recording interval. This beat was identified to be, typically, the largest or second-largest beat in the signal, where the spread in the voltage within the time window differed in a range of 0–0.2 mV. A color interpolation of voltage values between the recorded electrode positions of <7 mm was then applied to the geometry automatically by CARTO-3.

### 2.2. Voltage Mapping in Sinus Rhythm

The QRS complex was excluded from the window-of-interest during LA voltage mapping. LVA were defined using cut-off values for bipolar peak-to-peak voltage in SR of <0.5 or <1.0 mV (Jadidi et al., 2016; Rodríguez-Mañero et al., 2018). Areas demonstrating low voltage when mapped with the 20-pole Lasso catheter were confirmed using a contact force-sensing mapping catheter with a contact threshold of >5 g.

### 2.3. Voltage Mapping in Atrial Fibrillation

Patients underwent voltage mapping in AF using the sharp peak in the surface QRS as the reference. The QRS complex was then excluded from the window-of-interest during LA voltage mapping. To ensure the highest accuracy of electrogram criteria, >1,200 points were acquired per LA and rhythm. Respiratory gating was performed and the atrial geometry was acquired at high adjustment settings (geometry acquisition by Lasso catheter was set to 18 on CARTO-3) to obtain the highest accuracy of the acquired atrial geometry. Presence and accurate localization of low voltage areas was confirmed by contact force-sensing catheters (>5 g). Bipolar low voltage zones were defined as <0.35 or <0.5 mV in AF, according to the findings in recent studies (Jadidi et al., 2016, 2020; Rodríguez-Mañero et al., 2018).

The voltage was defined as the peak-to-peak amplitude of a single AF beat. In AF, the window of interest was set to 90% of the mean AF cycle length in order to consider only a single AF beat. This beat was manually selected with special emphasis on having only a single depolarization wavefront (AF beat). The current voltage mapping software of CARTO-3.7 does not support automatic voltage mapping during AF. Use of the automatic CARTO-3 software for voltage mapping in AF may result in mapped sites without any underlying electrogram (including the isolelectric intervals only) or including multiple AF beats with inadequate peak-to-peak voltage measurements. Therefore, in the current study, AF voltage maps were acquired manually.

### 2.4. Analysis

To identify the correlation between the unipolar and bipolar maps, the sensitivity, and specificity were calculated. The bipolar map was considered as the “true condition” and the electrogram at each point of the unipolar map was classified depending on the unipolar voltage threshold. Points labeled as low voltage in the (ground truth) bipolar map were identified as true positive (unipolar voltage < threshold) or false negative (unipolar voltage > threshold) in the unipolar map. True negative and false positive classes were similarly defined for the points with a supra-threshold voltage in the bipolar map. The unipolar threshold was then varied between 0.1 and 4 mV, and a receiver operating characteristic (ROC) curve was created to identify the unipolar threshold which provides the best match to the bipolar map for each patient and rhythm.

The relationship between unipolar and bipolar voltage was further explored by examining the percentage of points on the maps, which were classified the same in both cases (low or high voltage). This analysis was performed based on the voltage map provided by CARTO-3 (interpolated map data). The data from the electrograms at the mapping sites were used directly to analyze the effect of inter-electrode spacing.

For each patient, the best unipolar threshold corresponding to a specific bipolar threshold was identified using the ROC curve. Using one common unipolar threshold for all patients rather than an individual threshold for each patient was evaluated and analyzed by calculating the percentage of points that matched in unipolar and bipolar.

Regional differences in the voltage have been found in patients, with the anteroseptal LA wall and roof displaying the common and most extensive LVA, followed by the posterior LA wall (Marcus et al., 2007; Müller-Edenborn et al., 2019). To examine what effect the regional differences may have on the correlation between unipolar and bipolar LVA, each LA was split into 11 anatomical regions. The regions are as follows: orifices to the four pulmonary veins (LIPV, LSPV, RIPV, RSPV), the region around the mitral valve (MV), the left atrial appendage (LAA), the anterior, posterior, and lateral wall, the roof, and the septum. The percentage of points which matched between the unipolar and bipolar map were calculated for each anatomical region, using the bipolar threshold of 0.5 mV in SR and 0.35 mV in AF and the best corresponding unipolar threshold for each patient, which range between 0.62 and 1.1 mV (SR) and 0.45 and 0.99 mV (AF).

A factor that may influence the correlation between bipolar and unipolar mapping is the level of low voltage substrate in patients. Therefore, the 28 patients were split into four subgroups depending on the extent of low voltage (<0.5 mV during SR in the bipolar map). The low voltage substrate extent was then determined as the percentage of the surface area. Each patient was categorized into one of the four groups: stage I (<5%), II (≥5% to <20%), III (≥20% to <30%) and IV (≥30%) as suggested by Oakes et al. (2009) and Yamaguchi et al. (2018). The match between unipolar and bipolar classification was then calculated for each category, in both rhythms.

The distance between the bipolar electrodes is known to affect the bipolar signals (voltage increases as the distance increases) (Beheshti et al., 2018). Several studies have examined this effect concerning the influence it may have on the identification of LVA (Anter et al., 2015; Mori et al., 2018). Thus, the data provided by CARTO-3 were split into three groups: (1) containing only information from the 2 mm electrode distances, (2) only 6 mm distances, and (3) containing both. The percentage of points that matched between unipolar and bipolar was then calculated, and the paired-sample *t*-test was used to calculate if the difference between the three sets was significant.

## 3. Results

### 3.1. Patient Characteristics

Twenty-eight patients (66 ± 7 years old, 46% male, 82% persistent AF, 32% redo-procedures) underwent high-density (>1,200 sites, with a mapping density of 17 ± 7 sites per cm^2^ in SR and 22 ± 11 per cm^2^ in AF, using a 20-polar 2-6-2 mm-spaced Lasso, CARTO-3) voltage mapping in SR and AF prior to PVI. Further details on patients' characteristics are provided in Table 1. Additionally, information on the electro-anatomical mapping across all patients is provided in Table 2.

**Table 1:** Patient clinical demographics.

**Patient characteristics**	**Total = 28**
Rhythm at presentation (AF, %)	21 (75)
Persistent AF (%)	23 (82)
Age	66 ± 7
Male, *n* (%)	13 (46)
BMI (kg/m^2^)	28 ± 4
Weight (kg)	84 ± 13
LVEF (%)	54 ± 10
LA diameter (AP, mm)	46 ± 5
IVSEDD (mm)	10 ± 2
SHD (%)	12 (43)
CHA_2_DS_2_-VASc score	2.2 ± 1.8
Hypertension (%)	16 (57)
LV systolic dysfunction (<45%)	9 (32)
Diabetes (%)	4 (14)
Renal failure (GFR <50 ml/min, %)	8 (29)
History of stroke (%)	1 (3.6)
Coronary artery disease (%)	3 (11)
Antiarhythmic therapy except betablocker (%)	14 (50)
Beta blocker therapy (%)	21 (75)
Amiodarone (%)	6 (21)
Flecainide (%)	5 (18)
Sotalol (%)	1 (3.5)
Dronedarone (%)	2 (7)
Redo procedure for AF	9 (32)

*LVEF, left ventricular ejection fraction; SHD, structural heart disease; LV, left ventricular; BMI, body mass index; GFR, glomerular filtration rate*.

**Table 2:** Mapping information of patients included in the study.

**Electro-anatomical mapping**	**SR**	**AF**
Low voltage surface area [cm^2^ (%)] (SR and AF <0.5 mV)	23 ± 23 (31 ± 30)	42 ± 26 (52 ± 30)
Map points (pts)	1,536 ± 608	1,978 ± 925
Map points after processing (pts)	1,200 ± 632	1,639 ± 754
Bipolar voltage (mV)	1.15 ± 0.67	0.54 ± 0.22
Unipolar voltage (mV)	1.44 ± 0.71	0.73 ± 0.23

### 3.2. Spatial Distribution of Left Atrial Low Voltage Areas in Unipolar vs. Bipolar Mapping

The three-dimensional distribution patterns of the bipolar vs. unipolar LVA were highly concordant for all analyzed voltage thresholds and in all patients. 90 ± 5 and 85 ± 5% of mapped sites in SR and AF, respectively, were concordantly classified as low or high voltage both in bipolar vs. unipolar mapping mode. Discordant mapping sites located to the border zone of LVA.

**Figure 1:**
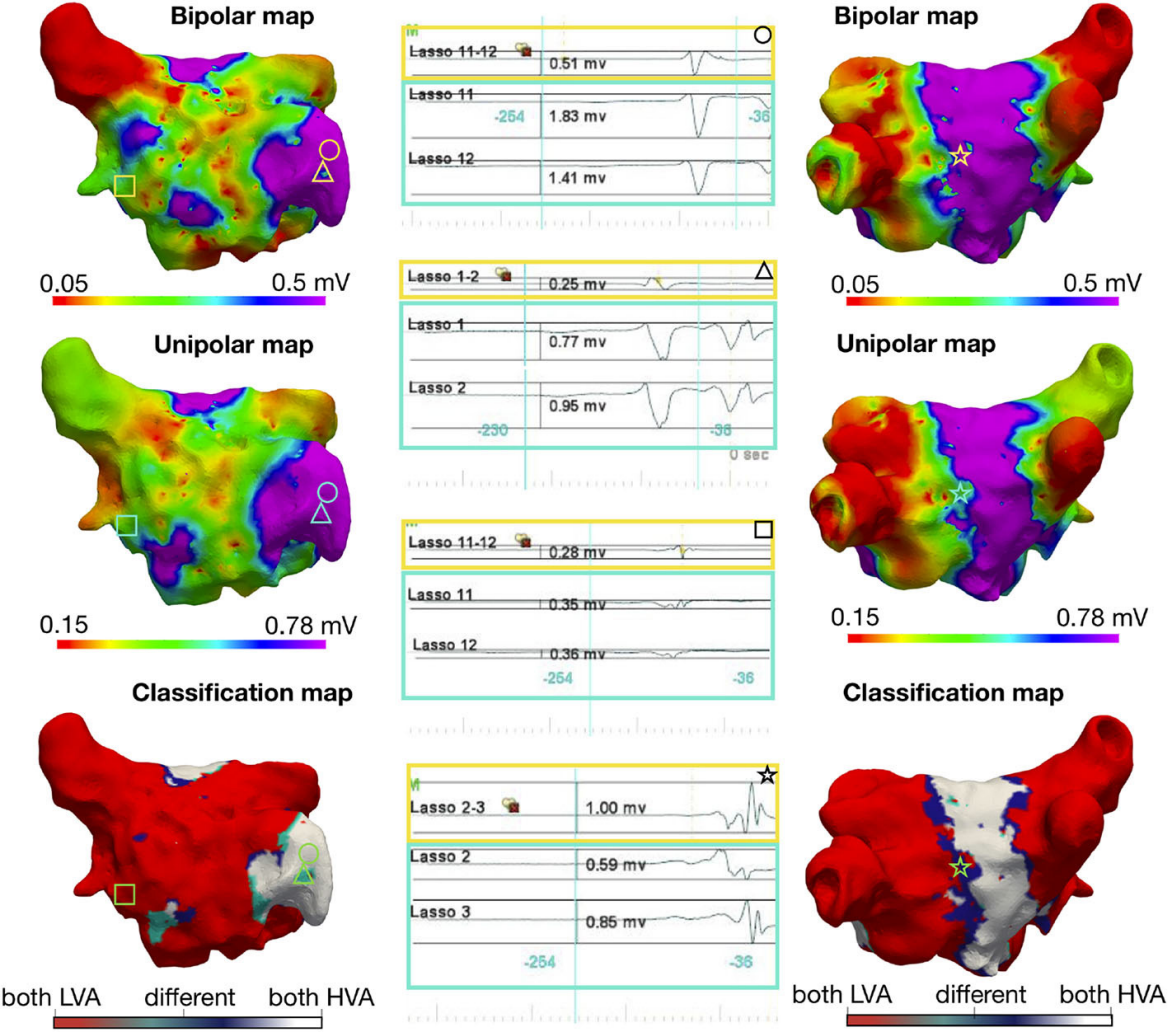
Voltage mapping in sinus rhythm: Three-dimensional distribution of bipolar and unipolar LVA in a patient during SR with a bipolar threshold <0.5 mV. The top row indicates the bipolar map, the middle row shows the unipolar map, and the bottom row is a map of the point by point comparison between the two maps: if both points are below their indicated voltage thresholds (LVA), the point is colored red, if both are above [high voltage areas (HVA)] in white and with different classification (bipolar low voltage, unipolar high voltage) in dark-blue and (bipolar high voltage, unipolar low voltage) in turquoise. In the left column, the anterior view is shown and on the right the posterior view. Each geometric shape represents a point on the map where the corresponding signal is shown in the middle column. The upper unipolar threshold values were obtained by determining which threshold provided the best match of points between unipolar and bipolar. For visualization, the lower value was manually optimized to obtain the best visual match.

**Figure 2:**
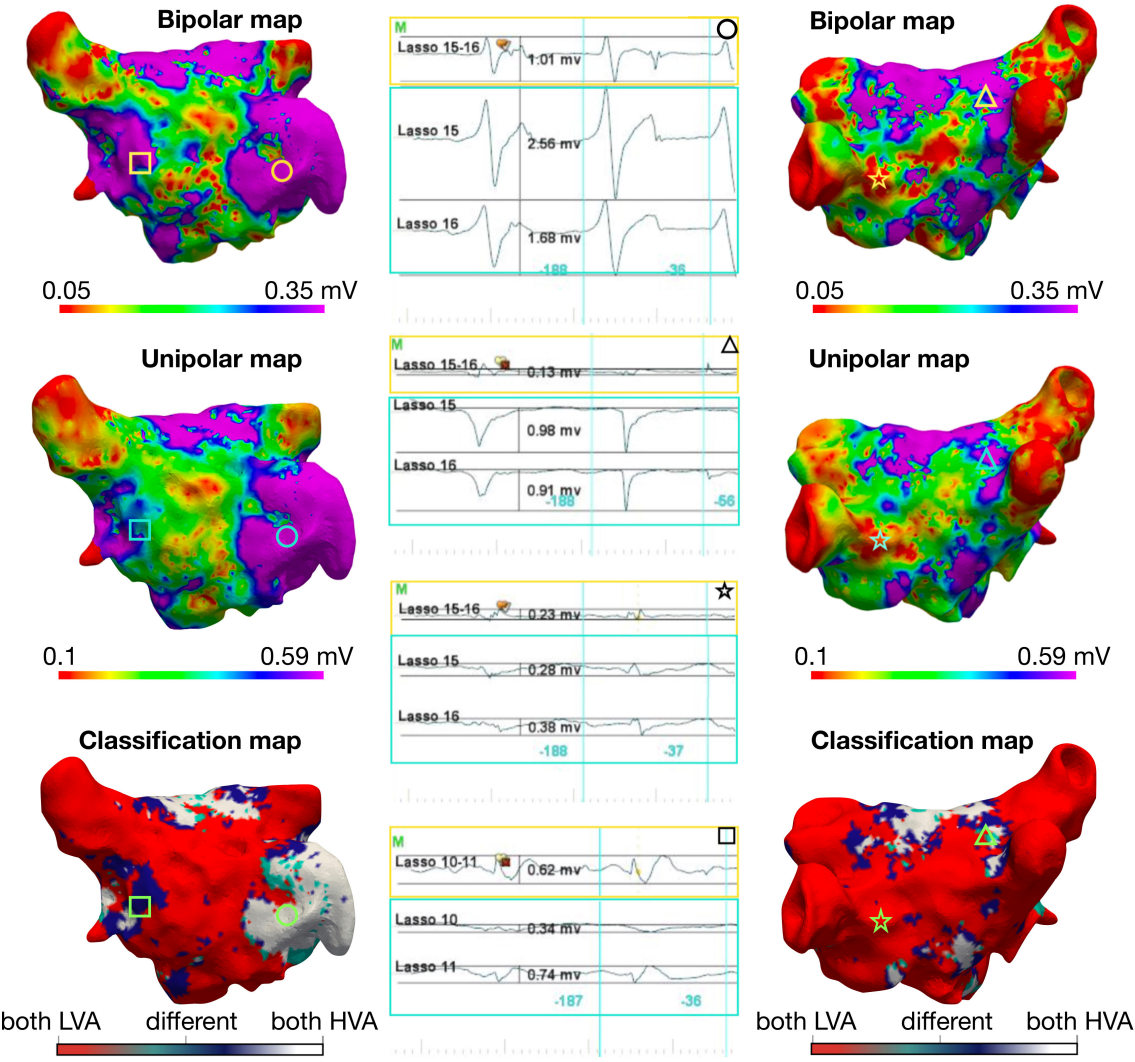
Voltage mapping in atrial fibrillation: Three-dimensional distribution of bipolar and unipolar LVA in the same patient as Figure 1 during AF with a bipolar threshold <0.35 mV. The top row indicates the bipolar map, the middle row shows the unipolar map, and the bottom row is a map of the point by point comparison between the two maps: if both points are below their indicated voltage thresholds (LVA), the point is colored red, if both are above [high voltage areas (HVA)] in white and with different classification (bipolar low voltage, unipolar high voltage) in dark-blue and (bipolar high voltage, unipolar low voltage) in turquoise. In the left column, the anterior view is shown and on the right the posterior view. Each geometric shape represents a point on the map where the corresponding signal is shown in the middle column. The electrogram marked with a triangle is an example of “voltage cancelation” in the bipolar recording (0.13 mV), while the voltages on both unipolar recordings are high (0.98 and 0.91 mV); this occurs because the unipolar electrograms have the same shape and same timing and their subtraction results in a near-zero bipolar voltage value.The upper unipolar threshold values was obtained by determining which threshold provided the best match of points between unipolar and bipolar. The lower value was selected manually to obtain the best visual match.

Figure 1 illustrates the three-dimensional distribution patterns of the bipolar vs. unipolar LVA for a single patient with electrogram examples. LVA were found at the same positions in the unipolar and bipolar maps using a unipolar threshold of 0.78 mV when the bipolar threshold was 0.5 mV. The classification map, in Figure 1 lower panels, identifies mapping sites where the maps disagree with regard to LVA in unipolar vs. bipolar mode. Such areas with different classification of LVA were mostly located at border zones, where the myocardial voltage amplitudes are changing from low to high voltage. The difference between the selected threshold value to the voltage values of miss-classified electrograms was found to be 0.3 ± 0.1 mV. Figure 2 shows similar results for bipolar vs. unipolar voltage maps acquired during AF. Additionally, the three-dimensional distribution patterns of LVA in unipolar and bipolar voltage maps are illustrated for all 28 patients in Supplementary Figures 1, 2. In these figures, the Pearson correlation coefficient between the bipolar and unipolar voltage maps prior to applying a threshold is shown for each patient. For both rhythms the correlation was found to be 0.88 ± 0.05, indicating a strong positive correlation between the mapping modalities independent of a specific threshold.

### 3.3. Spatial Correlation Between Unipolar vs. Bipolar Voltage Mapping—Regional Analysis

**Figure 3:**
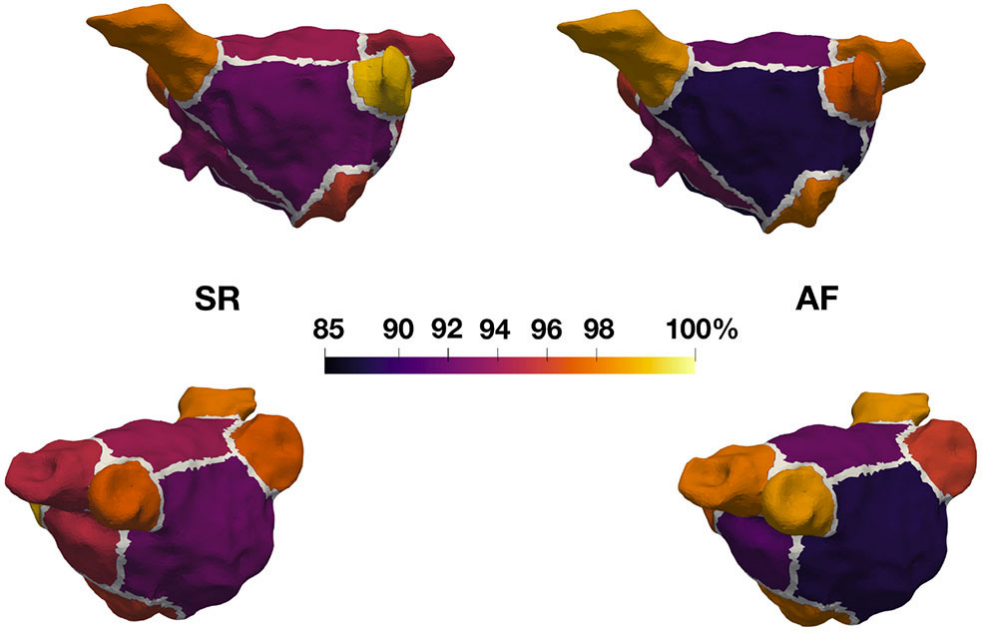
Regional agreement of unipolar and bipolar substrate classification. Eleven anatomical regions were annotated in all 28 patient geometries and analyzed in SR and AF. Each region visualized on this example geometry is colored according to the mean percentage of concordantly categorized mapping sites (unipolar vs. bipolar voltage mapping) with regard to LVA.

The overall three-dimensional distribution patterns of LA LVA during SR and AF (Supplementary Figures 1, 2) reveal a very high spatial concordance with very similar localization of LVA when comparing bipolar and the best correlated unipolar voltage thresholds. Further detailed regional quantification of the amount of concordant LVA classification between unipolar vs. bipolar voltage mapping is reported in Figure 3. The figure shows regional correlation results when splitting the LA into 11 anatomical regions. The mean percentage of concordantly categorized electrograms for each anatomical region divided by the number of electrograms within the same region (across all patients) is presented on an example geometry. Additionally, the mean and standard deviation values for each region can be seen in Table 3. For both rhythms, the LAA was one of the regions showing the highest agreement between the unipolar and bipolar voltage maps with 98% match in SR and 95% in AF. The pulmonary veins also display high agreement (93–96% SR and 94–97% AF). A slightly lower but still high regional similarity between the unipolar and bipolar maps was found within the body of the LA: LA posterior wall, anterior wall and the septum (90, 90, and 91% in SR and 87, 87, and 91% in AF). The high regional correlation between unipolar and bipolar voltage maps is well-reflected by the distribution of LVA patterns on the high-density interpolated electro-anatomical voltage maps (Supplementary Figures 1, 2). Independently of the underlying rhythm, the three-dimensional localization of the arrhythmogenic LVA is highly concordant and designates the same regions as potential ablation targets in unipolar and bipolar voltage mapping mode.

**Table 3:** Mean and standard deviation for the percentage of points that match in different anatomical regions of the LA [bipolar threshold of 0.5 mV in SR and 0.35 mV in AF and the best corresponding unipolar threshold for each patient, which range from 0.62 to 1.1 mV (SR) and 0.45 to 0.99 mV (AF)].

**Anatomical region**	**SR (%)**	**AF (%)**
LIPV	95 ± 6	97 ± 6
LSPV	93 ± 6	96 ± 3
RIPV	96 ± 6	97 ± 4
RSPV	95 ± 6	94 ± 7
MV	94 ± 5	96 ± 6
LAA	98 ± 3	95 ± 7
Anterior wall	90 ± 7	87 ± 8
Posterior wall	90 ± 8	87 ± 10
Lateral wall	93 ± 6	89 ± 7
Roof	92 ± 5	90 ± 7
Septum	91 ± 7	91 ± 7

### 3.4. The High Correlation Between Unipolar and Bipolar Voltage Maps Is Independent of the Underlying Extent of Low Voltage Substrate

The LA voltage maps of all 28 patients were categorized according to the extent of their LA low voltage substrate <0.5 mV in SR, as mentioned in section 2: seven patients in stage I, eight in stage II, five in stage III, and eight in stage IV. The low voltage surface area in SR at <0.5 mV bipolar threshold (mean ± standard deviation) in each group was: stage I: 3 ± 2%, stage II: 12 ± 5%, stage III: 27 ± 4%, stage IV: 37 ± 4%. The percentage of points that matched in the bipolar and unipolar voltage maps for patients with different levels of low voltage substrate is shown in Supplementary Figure 3. For all low-voltage substrate stages in both rhythms, the percentage of mapping points that matched was >85% (mean). The maximum difference between categories was 4%.

### 3.5. Specific Unipolar Voltage Thresholds Are Associated With High Correlation Between Unipolar vs. Bipolar Low Voltage Substrate

**Figure 4:**
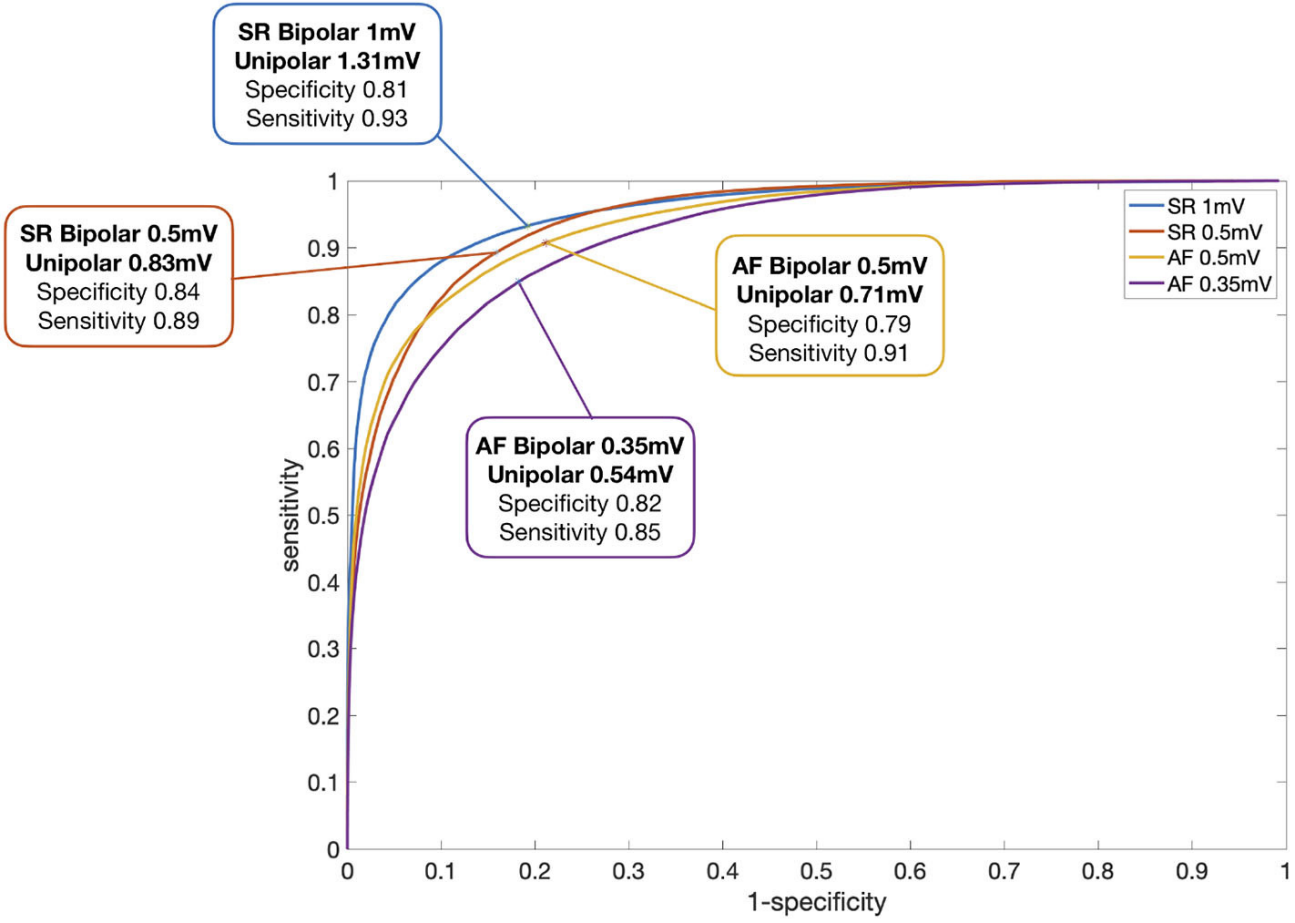
ROC curve regarding identification of LVA for each rhythm and bipolar threshold. Each line represents the sensitivity and specificity for varying unipolar thresholds for the four different bipolar voltage thresholds that are reported in literature for SR and AF. The optimal unipolar thresholds for the corresponding bipolar thresholds were found for each patient and the median value of these patient specific thresholds is given in the figure.

Figure 4 illustrates the ROC curves for the optimal unipolar voltage thresholds that result in the highest concordance of electrogram classifications to high and low voltage areas in both unipolar and bipolar maps for four previously described bipolar voltage thresholds. For each rhythm and voltage threshold, there was a high percentage of agreement (sensitivity and specificity >80% in all cases). The highest sensitivity and specificity values (93, 81%) were obtained when comparing the maps in SR using a bipolar threshold of 1 mV and a unipolar threshold of 1.31 mV. Sensitivity and specificity values were as follows for the other cases: SR with bipolar threshold of 0.5 mV (unipolar threshold 0.83 mV, 89%, 84%), AF 0.5 mV (unipolar 0.71 mV, 91%, 79%), and AF 0.35 mV (unipolar 0.54 mV, 85%, 82%).

**Figure 5:**
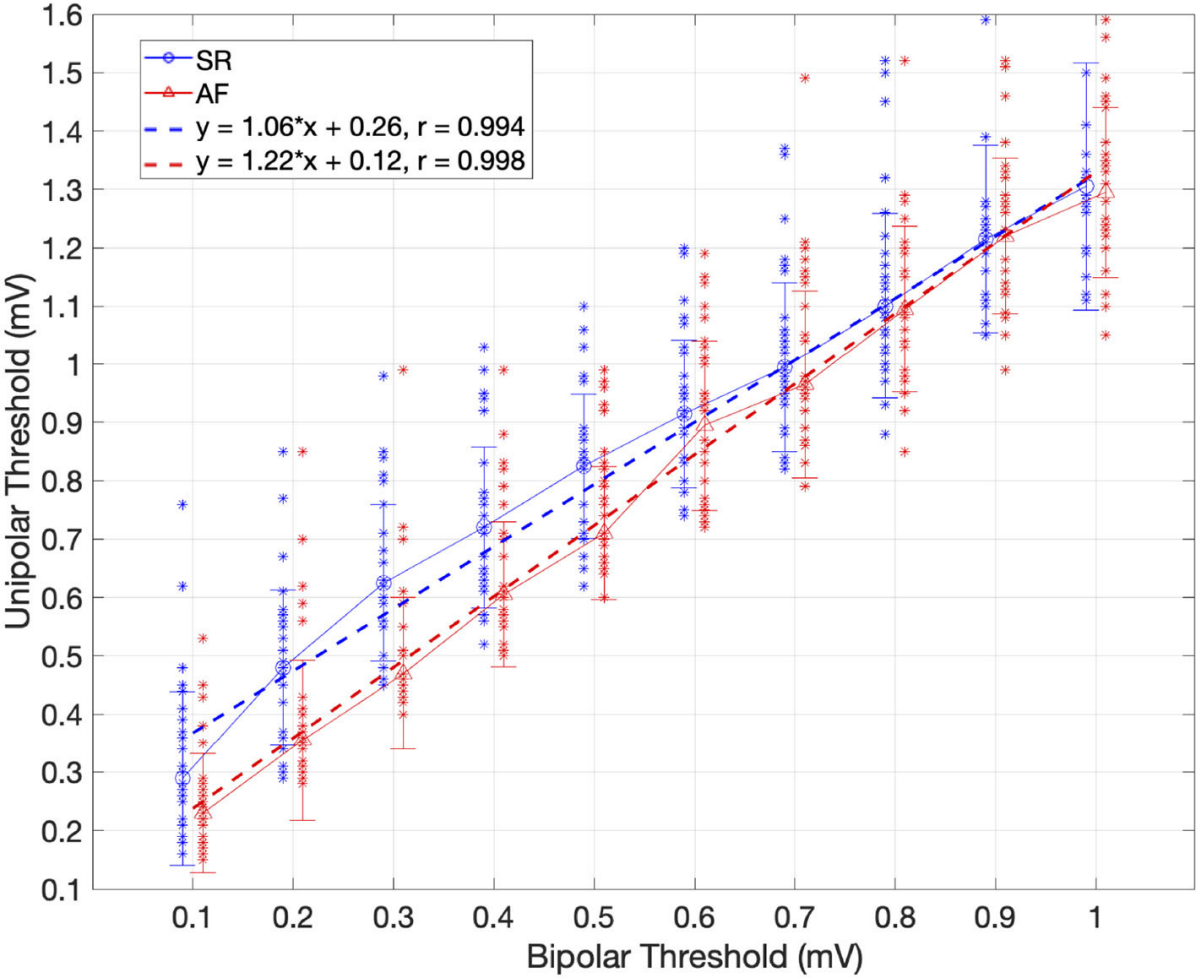
Relationship between the bipolar and unipolar threshold in SR (blue) and AF (red). The unipolar threshold with highest concordance to the bipolar map is shown for different bipolar thresholds and each individual patient (blue and red dots for SR and AF, respectively). Standard deviation is represented by bars. The optimal unipolar threshold is identified as the optimal point on the ROC curve of each bipolar threshold using all patients. Linear regression shown as dotted lines, depending on the rhythm (SR blue, AF red). Pearson correlation coefficient (r) is provided. For every unipolar threshold, >89% of points matched in SR and >86% in AF.

To further examine the relationship between the bipolar and the unipolar voltage threshold, a set of SR and AF ROC curves were created for each bipolar threshold between 0.1 and 1 mV, in steps of 0.1 mV. In this way, the optimal unipolar threshold for each bipolar threshold was identified, which gave the highest classification match between the mapping modalities. The unipolar threshold values were then plotted against the bipolar threshold values, as shown in Figure 5. Linear regression yielded the following relations: *y* = 1.06*x* + 0.26*mV* for SR and *y* = 1.22*x* + 0.12*mV* for AF, where y is the unipolar threshold and x is the bipolar threshold in mV. The correlation coefficient between the two variables (bipolar and unipolar threshold) shows a strong positive correlation for both rhythms (0.994 in SR and 0.998 in AF). However, as there is spread between values for individual patients, the y-intercept cannot be as reliably determined as the slope of the relation. A simplified calculation of the corresponding unipolar threshold is provided by the addition of 0.3 vs. 0.2 mV to the bipolar voltage threshold in SR vs. AF, respectively (unipolar threshold error <0.07 mV in the bipolar threshold range between 0.1 and 1 mV).

### 3.6. Comparison of Unipolar and Bipolar Low Voltage Areas Using a Common vs. a Patient-Specific Unipolar Threshold

**Figure 6:**
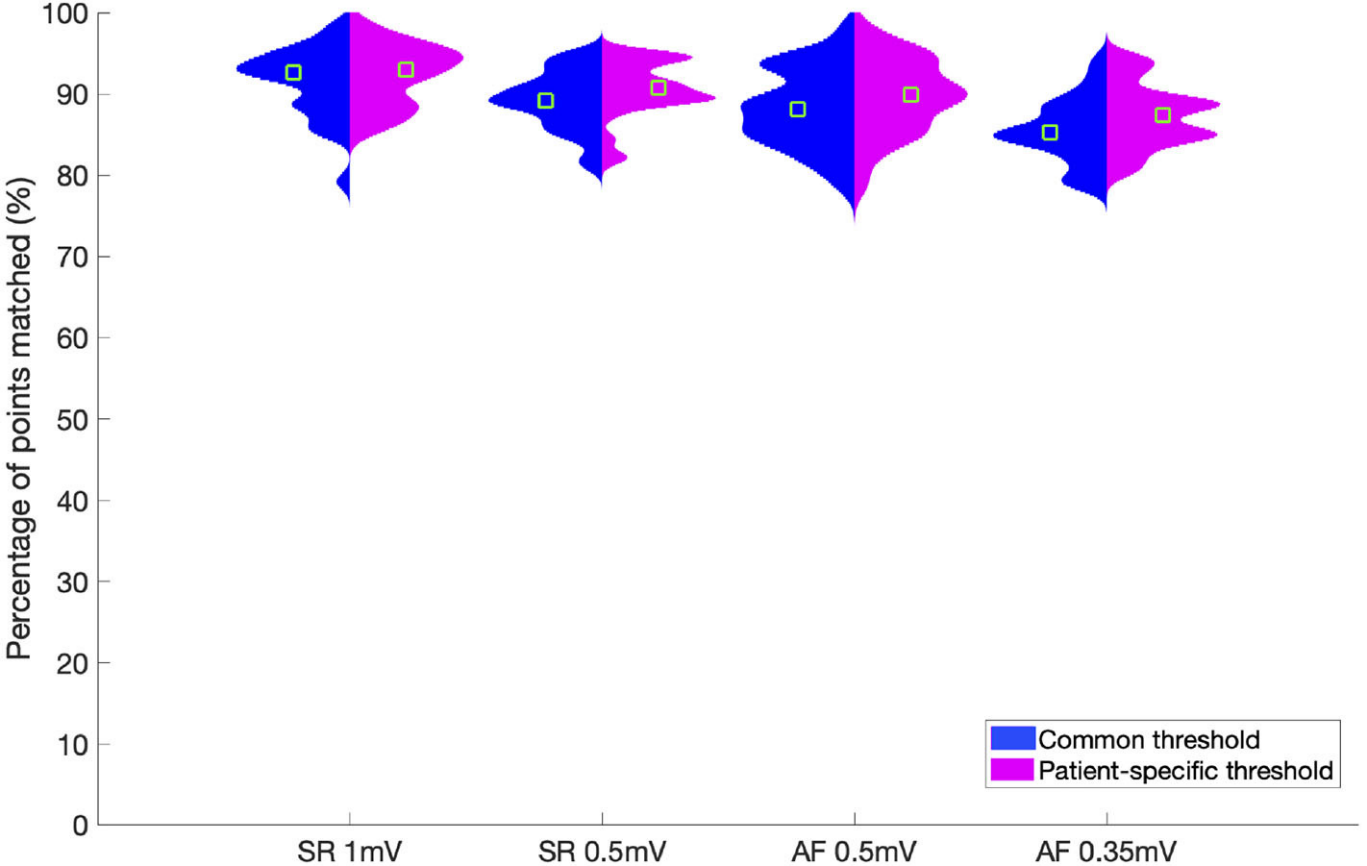
Violin plot showing the percentage of points which match between unipolar and bipolar classification. The results are shown for each rhythm and corresponding bipolar threshold over all patients. Results for a common unipolar threshold for all patients are shown in blue. Results for patient-individual unipolar thresholds are shown in pink. The green squares indicate the median value for each set.

The difference between using one universal unipolar threshold for all patients (Figure 4), vs. patient-specific thresholds were evaluated to assess the variability in identifying low voltage areas. Figure 6 illustrates that the increase in electrogram classification accuracy with the use of patient-specific vs. a common unipolar threshold is marginal (1–2%). For SR with a bipolar threshold 1 mV, the improvement in substrate classification changed from median of 92.7% using a common unipolar threshold to 92.9% using a patient-specific unipolar threshold. Using a Wilcoxon rank sum test, the difference between using one common threshold or individual thresholds was found to be not significant for either rhythm or bipolar threshold used. From Figure 5 the best unipolar threshold for each patient can be seen in a range of 0.3 mV. However, the similarity between unipolar and bipolar mapping remains high regardless if a common threshold is used or a patient-specific one. This shows that within each patient, a classification margin exists of at most, ± 0.15 mV from the common threshold. Therefore, only a few data points have voltage values within this margin and are affected by changing the threshold in this range.

### 3.7. Impact of Bipolar Inter-electrode Distance on the Identified Low Voltage Substrate

**Figure 7:**
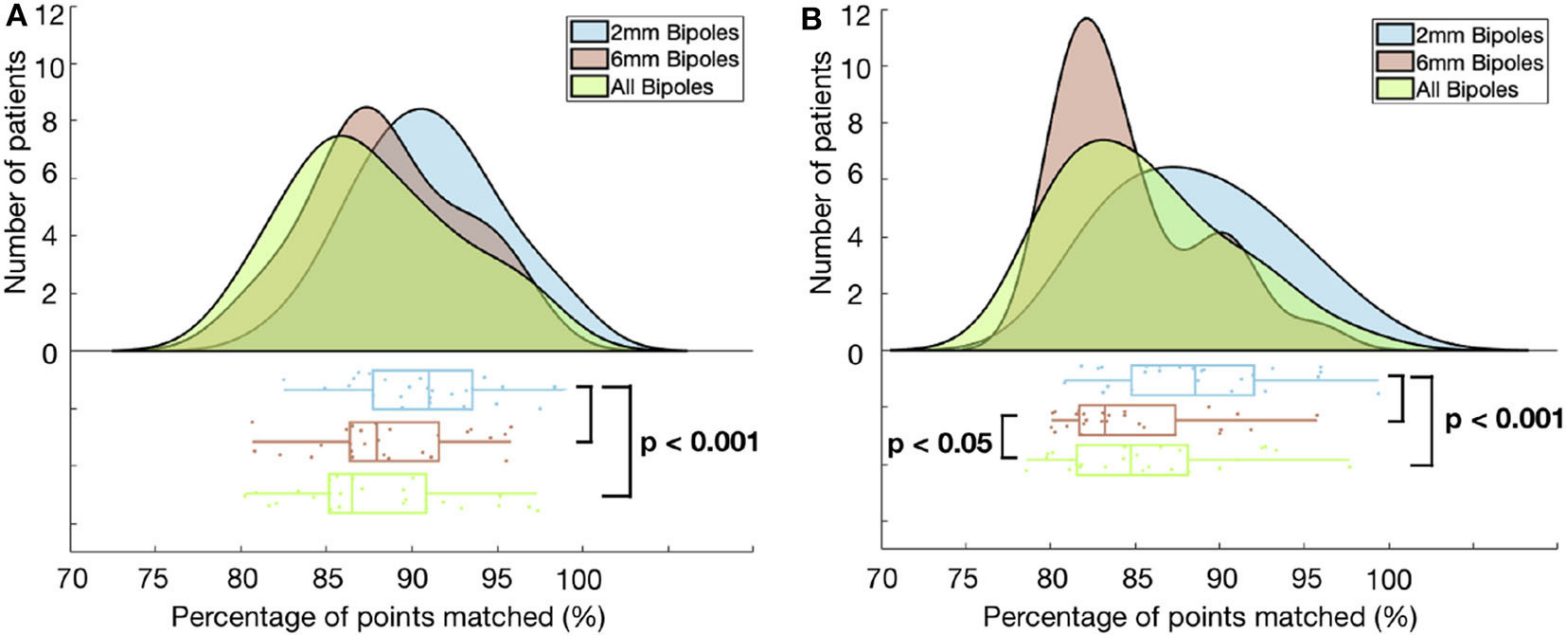
Histogram and corresponding boxplot showing the percentage of points matched in SR **(A)** and AF **(B)** for each of the three electrode distance categories. Each category is shown in a different color: 2 mm (blue), 6 mm (red), all (green). *p*-value calculated with paired-sample *t*-test.

We analyzed the impact of different inter-electrode distances on bipolar LVA distribution and its correlation to the corresponding unipolar voltage map. Therefore, LA electrograms were analyzed using each bipolar electrode distance (2, 6 mm and both) separately (see Figure 7). For both SR and AF, we found that when considering only the small bipoles (2 mm), 3–6% more mapping sites matched than when using the large bipoles (6 mm) or both together. Although the differences in LVA categorization remained small, they are statistically significant. Agreement of LVA categorization using the small bipoles was higher (SR 91%, AF 89% of mapping sites) than for mapping with the large or all bipoles in SR (88 and 87%) and in AF (83 and 85%) (*p* <0.001 for all cases). The optimal common unipolar threshold for each bipole distance is given in Table 4. The unipolar threshold for the all bipoles is slightly higher than reported in Figure 5 (0.87 vs. 0.83 and 0.64 vs. 0.54 mV). This discrepancy is because this analysis was performed on the electrode signals directly rather than on the interpolated voltage map data. Only a 1% discrepancy in the median percentage of points that matched was seen when using the measured electrograms directly than the interpolated map data.

**Table 4:** Rhythm-specific unipolar threshold corresponding to each bipolar threshold for each group of bipolar electrode distance.

	**SR Threshold (mV)**		**AF Threshold (mV)**	
**Distance (mm)**	**Bipolar**	**Unipolar**	**Bipolar**	**Unipolar**
2	0.5	1.09	0.35	0.76
6	0.5	0.72	0.35	0.52
All	0.5	0.87	0.35	0.64

The best unipolar threshold for bipolar voltage maps (SR <0.5 mV) acquired with the small 2 mm distant electrode pairs was found to be higher (1.09 mV SR and 0.76 mV AF) than when using the large 6 mm distant electrode pairs (0.72 and 0.52 mV). Supplementary Figure 4 shows the relationship between the bipolar and unipolar threshold when only using 2 mm bipoles (Supplementary Figure 4A) and 6 mm (Supplementary Figure 4B).

## 4. Discussion

### 4.1. Main Findings

The current study on bipolar vs. unipolar voltage mapping reveals three main findings:

1. There is a high correlation in the spatial distribution of uni- and bipolar low voltage areas in sinus rhythm and atrial fibrillation.
2. Over 90% of LA electrograms are concordantly classified as high or low voltage using uni- or bipolar mode independently of the selected bipolar threshold. The remaining discordant electrograms locate to low voltage border zones without change of identified LVA.
3. Bipolar electrode distance has little impact on the agreement between unipolar and bipolar voltage maps.

Previous studies have suggested that diseased tissue can be identified in SR as bipolar voltage areas below 0.5 or 1 mV and similarly for AF with voltages below 0.35 or 0.5 mV (Jadidi et al., 2016; Yang et al., 2016; Rodríguez-Mañero et al., 2018). Our results reveal that for any given bipolar voltage threshold, a universal unipolar voltage threshold can be found that results in a highly similar unipolar voltage map with a spatial distribution of LA LVA that corresponds to the bipolar LVA. For a bipolar threshold of 0.5 mV in SR, a unipolar threshold of 0.83 mV was optimal. In AF, a unipolar threshold of 0.54 mV was found for a bipolar threshold of 0.35 mV. More generally, the unipolar threshold for identifying the same low voltage regions can be obtained by applying a linear transformation to the bipolar threshold being used. This threshold is dependent on the size of the bipolar electrode spacing (Figure 5, Supplementary Figures 4A,B). The linear regression lines describing the relationship differ between SR and AF, where lower unipolar thresholds were identified in AF than in SR. One possible reason for this can be that in AF, the propagation arrives at the electrodes from different directions. Therefore, the lower voltage values due to the catheter orientation are reduced in the bipolar AF map. Since there are fewer low voltage points, a lower unipolar threshold is needed to match the smaller bipolar low voltage areas.

Using patient-individual thresholds increases the agreement between unipolar and bipolar maps by up to 2%. In order to identify the optimal patient-specific unipolar threshold to a pre-selected bipolar threshold in clinical practice, the bipolar and unipolar voltage maps can be visualized side by side and the unipolar threshold can manually be adapted to a level at which the distribution of LVA show the best concordance/correlation to the bipolar voltage map. Our study revealed a very similar distribution of LVA during both mapping modes. Therefore, the utility of a unipolar voltage map beyond the bipolar map has to be assessed in future studies, eventually evaluating these correlations for other mapping catheters.

These high levels of correlation between the unipolar and bipolar map were seen in all four patient subgroups defined by the low voltage substrate extent with only little variation in accuracy of 5% between subgroups. Therefore, regardless of the low voltage substrate extent in a patient, the LVA will be identified in the same locations in both the bipolar and unipolar map.

Previous studies have shown that AF driver sites with acute AF termination frequently occur within LVA in bipolar mapping (Jadidi et al., 2016, 2020). Considering our findings, atrial sites displaying low voltage in the bipolar map will also display these regions in the unipolar map, thus indicating that an important criterion for AF source localization during high-density Lasso mapping is reduced electrogram voltage irrespective of the mapping modality (uni-/bipolar).

### 4.2. Impact of Electrode Spacing and Atrial Anatomical Region on Voltage Mapping

In this study, we split the voltage information into groups depending on the bipole pair that the signals were obtained from. Small bipolar inter-electrode distance (2 mm) yielded a significantly higher percentage of points being matched correctly in bipolar vs. unipolar voltage mapping, both in SR and AF. When using only the small bipoles, the signal collected from the bipolar pair is more localized, i.e., the region covered by the electrodes is smaller. The signal is less susceptible to influences of far-field, as presented by Takigawa et al. (2019). Therefore, mapping with the small distance electrode pairs improves the correlation between unipolar and bipolar mapping at 4% of mapping sites. Importantly, the optimal unipolar threshold has to be set to a higher value when bipolar mapping is done with small bipoles, resulting in larger LVA than mapping with large bipoles. In contrast, voltage maps acquired with large-spaced bipoles integrate high-voltage far-field signals from adjacent healthier myocardium and therefore, large-spaced bipolar maps under detect low voltage tissue. The unipolar voltage map, therefore, has to be set to a lower threshold to display the smaller LVA of the large-spaced bipolar map.

Recent studies revealed a preferential anatomical distribution of LA LVA that more frequently affects the LA antero-septal area, followed by the LA roof and LA posterior wall (Corradi et al., 2005; Marcus et al., 2007; Müller-Edenborn et al., 2019). Therefore, it is more likely that LVA are present in these regions. From our analysis of assessing the different LA regions separately, we see that some of the lowest similarity between bipolar and unipolar signals exist in the posterior wall (90% SR, 87% AF match), the anterior wall (90% SR, 87% AF), and the septum (91% SR, 91% AF). This finding can be explained by the higher rate of occurrence of LVA in these regions, as this also means that more border zones are present and mismatch between unipolar and bipolar classification mainly occurred in border zones (Figure 1). In other areas such as the pulmonary veins, we typically have low voltage across the entire region and, therefore, fewer border zones, which leads to less mismatch in these regions.

### 4.3. Possible Reasons for the High Similarity Between Unipolar and Bipolar Low Voltage Distribution Patterns

Our findings show a high agreement between the bipolar and unipolar mapping when using high-density multi-electrode mapping with a 20-pole Lasso catheter. However, from simulation studies, it is known that the angle of the bipolar electrode pair in relation to the propagation direction and the size and distance between the electrodes are known to affect the bipolar voltage amplitude (Schuler et al., 2013; Beheshti et al., 2018). A recent study has shown that for 2 and 6 mm distant electrodes in simulated and clinical data, the amplitude of the bipolar electrogram goes from a maximum value at 0° to the propagation of a planar wavefront to close to 0 mV at 90° (Gaeta et al., 2019). Therefore, one can hypothesize that the bipolar voltage map would not present the same information as the unipolar voltage due to the direction dependence. Despite these factors, in our study, the majority of points (90%) were consistently categorized as either low or high voltage in both unipolar and bipolar. Small differences, which are clinically irrelevant, only exist at the border zones of the low voltage to high voltage threshold. Therefore, it is crucial to understand why the majority of points on the map have the same classification in both maps when the bipolar electrograms are influenced by various factors of the catheter. Here we discuss potential reasons why the theory-based expectations about the effects of using a bipolar catheter are hardly met by what was empirically identified in this study.

The simulation studies show that when the electrode orientation is perpendicular to the propagation, the bipolar voltage is 0 mV. This situation is shown in Figure 2 by the electrograms marked with a triangle. However, the high overall correlation between the high-density unipolar vs. bipolar maps indicates that this situation rarely occurs in practice. One explanation may be the following: In the clinical setting of the 1–5 mm thick atrial wall with multiple layers of myocardial fibers, it may be extremely rare that the wavefront is uniformly parallel to the mapping bipole. Instead, the waves always contain some degree of curvature in the three-dimensional space, so that the electrodes do not receive the signal at the same time point, which would result in a voltage >0 mV. Thus, the effect of the bipolar orientation is likely to be less pronounced in clinical mapping settings than what is presented in idealized simulations with homogeneous tissue and (almost) perfectly planar wavefronts.

Another possible explanation for obtaining such a high correlation is due to the maps containing a high density of points. Thus, points in close proximity can be obtained from the catheter at various bipole orientations. Therefore, when interpolation of voltages is applied in the electro-anatomical maps, areas with low and high voltage points caused by the orientation of the catheter may result in an averaged value. Thus, some points which are affected by the catheter orientation may cancel out by near-by adjacent mapping points with different orientations to the wavefront. The use of interpolated vs. non-interpolated voltage maps yielded similar levels of agreement, suggesting that this effect does not notably contribute. During ongoing AF with changing wavefront directions, the direction dependency of the bipolar voltage maps should be further reduced (in comparison to regular rhythms).

Additionally, if the unipolar voltages at points in the diseased tissue are much smaller than the threshold or much larger than the threshold in healthy regions, then there is a substantial classification margin. This would result in the distortion of the bipolar voltages due to the unknown orientation of electrodes to still be within this margin and the classification to be the same for the bipolar and unipolar map. For the points in which the voltages are close to the threshold values, the classification is more susceptible to changes in the voltage due to the orientation or the distance between the electrodes. Furthermore, if the two electrodes used to calculate the bipolar voltage have slightly different distances to the endocardium, the bipolar voltage would be reduced. However, for unipolar voltages with a large amplitude, then this difference would cause the bipolar voltage to be reduced but not substantial enough to cause the value to fall below the threshold. In Figure 1, the bipolar voltage maps show more patchy/irregular areas than the unipolar map. This shows that the bipolar map may be affected by factors such as wavefront-to-bipole orientation in these areas. However, most regions are far enough from the threshold for the orientation of the catheter to affect the classification.

Finally, the unipolar threshold was identified to provide the best correlation between the unipolar and the bipolar map based on the standard bipolar threshold used in practice. This, however, may not lead to the best unipolar threshold for identifying true areas of fibrotic tissue. Future investigations will help to unravel which of the aforementioned potential reasons contribute most to the agreement between both mapping modalities. Nevertheless, this study has shown that with appropriate settings and the identical catheter, the same LVA can be identified when using the bipolar or unipolar map.

## 5. Limitations

In this study, CARTO-3 was used for electro-anatomic voltage mapping, with a Lasso catheter of 2 and 6 mm inter-electrode spacing. Since only one type of catheter was used, it may be that our results are not applicable when using other catheters or mapping systems. However, we expect similar results with high-density maps acquired using a PentaRay or OctaRay catheter, where the mapping conditions such as electrode size, inter-electrode distance, and the parallel orientation of bipoles to endocardium are similar as in the current mapping study. We aimed at having high-density maps with a sufficient number of data points across the entire atria. For some patients, more points were taken at specific areas of interest; therefore, the distribution of points was not always equal between patients. However, it was ensured that the mapping density was sufficiently high (17 and 22 mapping sites per cm^2^ for SR and AF maps, respectively) to allow for regional analysis of the voltage maps. In this work, only one atrial beat was considered per mapped LA site (without averaging of multiple consecutive beats). However, due to the high density of the acquired maps, the atrial tissue was characterized by numerous mapping points that were recorded within a short distance from each other and contributed to the final voltage distribution maps in CARTO-3.

## 6. Conclusion

Bipolar and unipolar voltage maps are highly correlated both in SR and AF. Both mapping modes identify the same atrial sites as low voltage substrate. Small differences in low voltage classification may occur at the border zone of low voltage areas, without relevant impact on their spatial distribution patterns. Voltage mapping using small (2 mm) bipoles slightly improves the agreement between unipolar and bipolar low voltage areas. While bipole orientation and inter-electrode spacing are theoretical confounders, their impact is not of clinical importance, when mapping is performed at high density with a 20-polar Lasso catheter.

## Data Availability Statement

The data analyzed in this study is subject to the following licenses/restrictions: To protect the safety of the patients, the data used for this study can not be provided. However, the figures within the article and Supplementary Material show detailed analyses for all patients used. Requests to access these datasets should be directed to: Deborah Nairn, deborah.nairn@kit.edu.

## Ethics Statement

The studies involving human participants were reviewed and approved by University Hospital of Freiburg Ethics Committee. The patients provided their written informed consent to participate in this study.

## Author Contributions

DN, HL, AJ, and AL designed the study and analyzed the results. HL and AJ collected the data. SS developed the automatic tool for sectioning the atria. DN carried out the data analysis and produced the initial draft of the manuscript. All authors critically revised the manuscript and approved the final submitted manuscript.

## Conflict of Interest

The authors declare that the research was conducted in the absence of any commercial or financial relationships that could be construed as a potential conflict of interest.
