# Supplementary material for: Comparison of Unipolar and Bipolar Voltage Mapping for Localization of Left Atrial Arrhythmogenic Substrate in Patients With Atrial Fibrillation
Source: Front Physiol. 2020 Nov 26;11:575846. doi: 10.3389/fphys.2020.575846 (PMC7726205; doi:10.3389/fphys.2020.575846)
Supplement: Supplementary file 1 [file Data_Sheet_1.PDF]

1 SUPPLEMENTARY TABLES AND FIGURES

1.1 Figures

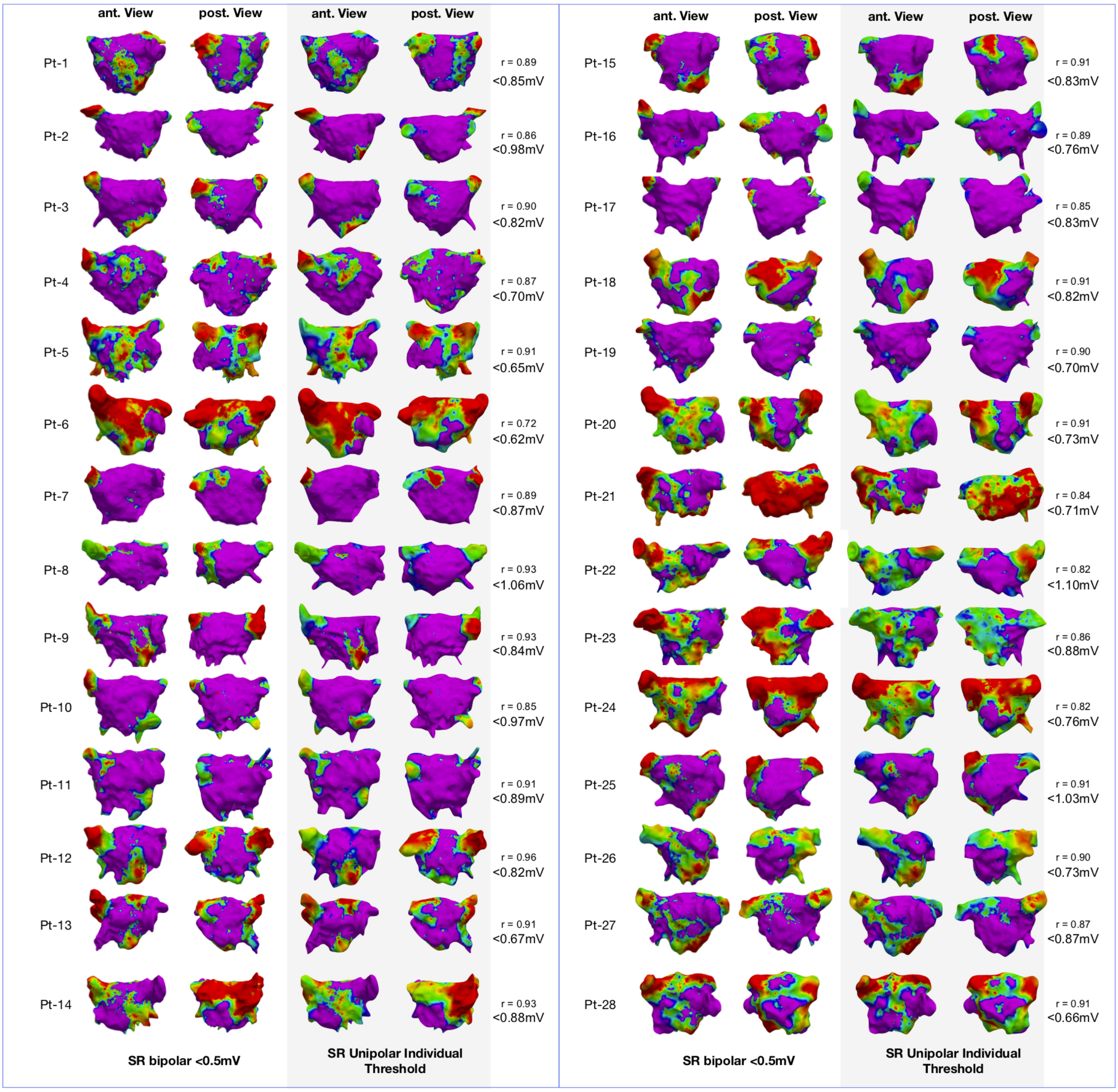

Figure S1. Three-dimensional distribution patterns of low voltage substrate in bipolar vs. unipolar voltage mapping mode for each one the 28 patients in SR with a bipolar threshold <0.5 mV. On the left side, patients 1-14 are shown, where the columns from left to right indicates: (1) the bipolar map (anterior view), (2) the unipolar map (anterior view), (3) the bipolar map (posterior view) and (4) the unipolar map (posterior view). On the right side the same is shown for patients 15-28. The voltage threshold of the unipolar maps were optimized for each patient to find the highest spatial overlap between LVS in bipolar vs unipolar maps. This optimal patient-specific unipolar threshold and the Pearson correlation coefficient is annotated at the right side of each row.

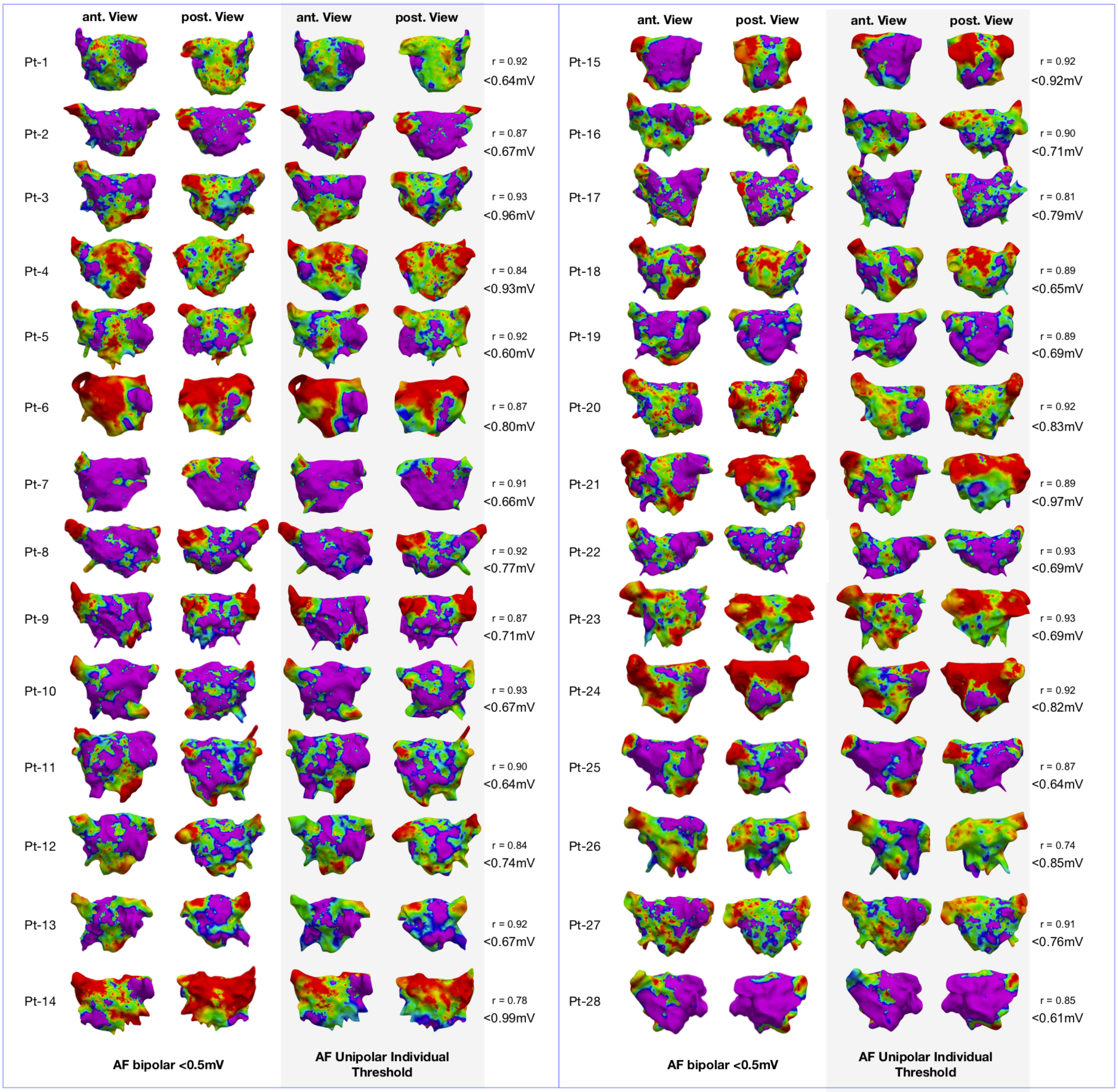

Figure S2. Three-dimensional distribution patterns of low voltage substrate in bipolar vs. unipolar voltage mapping mode for each one the 28 patients in AF with a bipolar threshold <0.5 mV. On the left side, patients 1-14 are shown, where the columns from left to right indicates: (1) the bipolar map (anterior view), (2) the unipolar map (anterior view), (3) the bipolar map (posterior view) and (4) the unipolar map (posterior view). On the right side the same is shown for patients 15-28. The voltage threshold of the unipolar maps were optimized for each patient to find the highest spatial overlap between LVS in bipolar vs unipolar maps. This optimal patient-specific unipolar threshold and the Pearson correlation coefficient is annotated at the right side of each row.

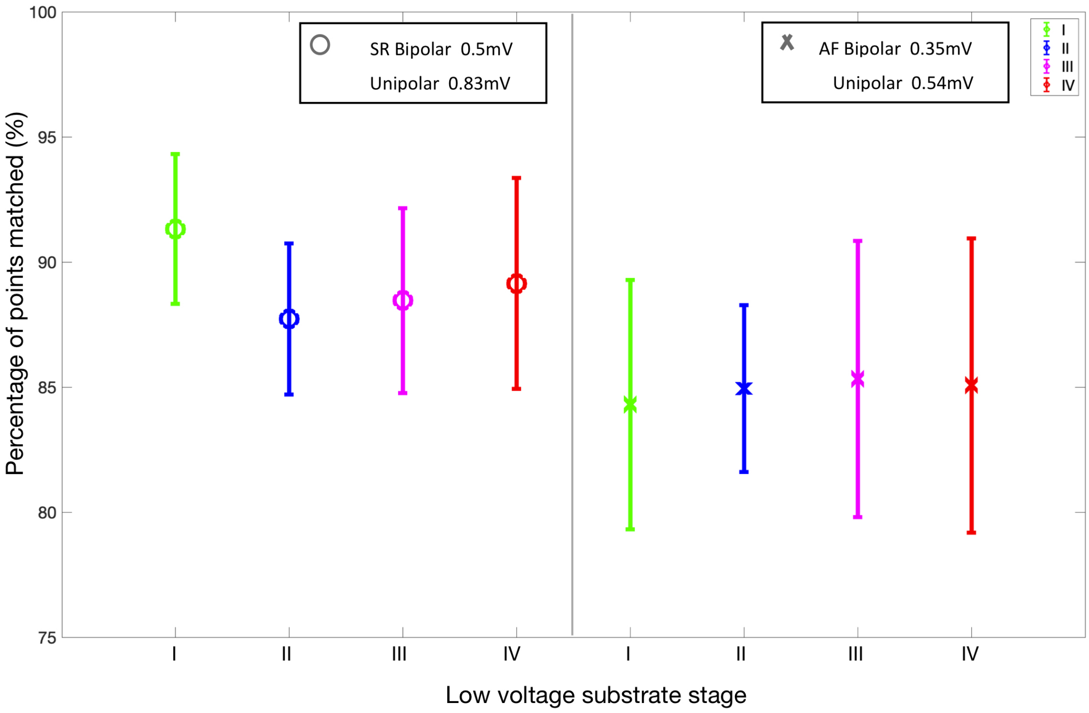

Figure S3. Mean and standard deviation of the percentage of points which match for the 4 different patient subgroups defined by the extent of low voltage substrate. Each color represents a subgroup (I-IV) in SR and AF for the given bipolar and unipolar thresholds as found in figure 4 of the main article.

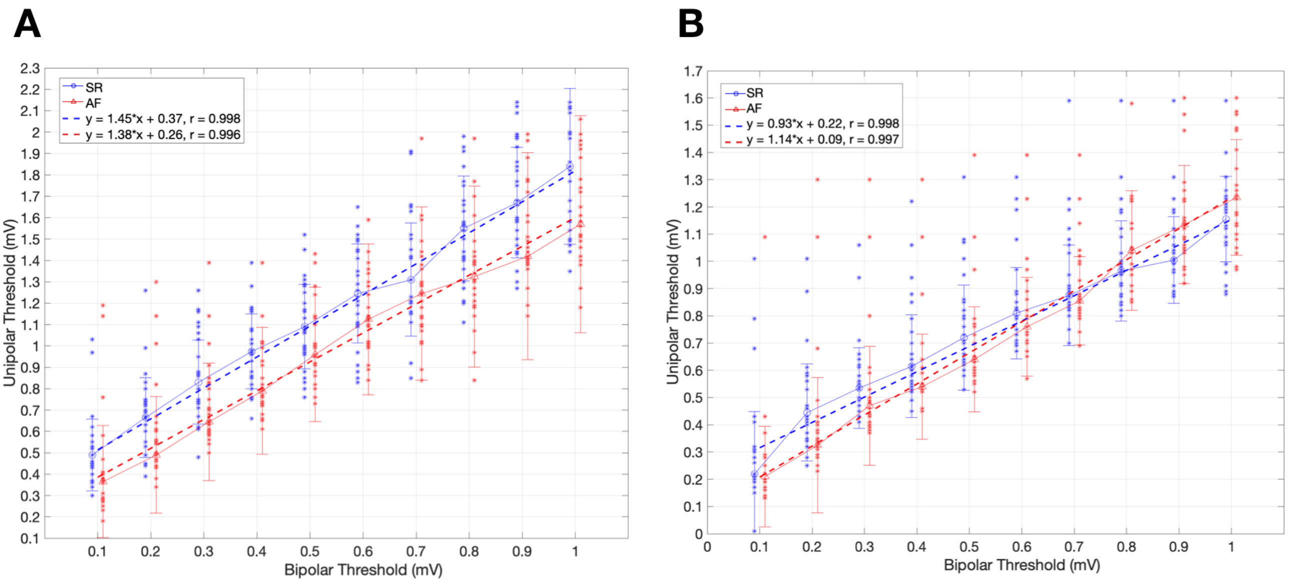

Figure S4. Relationship between the bipolar and unipolar threshold in SR (blue) and AF (red) using only 2mm bipoles (A) and 6mm (B). The unipolar threshold with highest concordance to the bipolar map is shown for different bipolar thresholds and each individual patient (blue and red dots for SR and AF, respectively). Standard deviation is represented by bars. The optimal unipolar threshold is identified as the optimal point on the ROC curve of each bipolar threshold using all patients. Linear regression shown as dotted lines, depending on the rhythm (SR blue, AF red). Pearson correlation coefficient (r) is provided.
